# Escin induces caspase-dependent apoptosis and autophagy through the ROS/p38 MAPK signalling pathway in human osteosarcoma cells *in vitro* and *in vivo*

**DOI:** 10.1038/cddis.2017.488

**Published:** 2017-10-12

**Authors:** Jian Zhu, Wei Yu, Bing Liu, Yitian Wang, Jianlin shao, Junjie Wang, Kaishun Xia, Chengzhen Liang, Weijing Fang, Chenhe Zhou, Huimin Tao

**Affiliations:** 1 Department of Orthopedics, 2nd Affiliated Hospital, School of Medicine, Zhejiang University, #88 Jie Fang Road, Hangzhou, Zhejiang 310009, PR China; 2 Orthopedics Research Institute of Zhejiang University, #88, Jiefang Road, Hangzhou 310009, PR China; 3 La Jolla Institute for Allergy and Immunology, La Jolla, CA, USA

## Abstract

Osteosarcoma is one of the most malignant neoplasms in adolescents, and it generally develops multidrug resistance. Escin, a natural mixture of triterpene saponins isolated from Aesculus hippocastanum (horse chestnut), has demonstrated potent anti-tumour potential *in vitro* and *in vivo*. In the present study, we found that escin inhibited osteosarcoma proliferation in a dose- and time-dependent manner. Additionally, escin-induced apoptosis was evidenced by the increased expression of caspase-related proteins and the formation of apoptotic bodies. Escin also induced autophagy, with elevated LC3, ATG5, ATG12 and Beclin expression as well as autophagosome formation. Inhibition of escin-induced autophagy promoted apoptosis. Moreover, p38 mitogen-activated protein kinases (MAPKs) and reactive oxygen species (ROS) were activated by escin. A p38 MAPK inhibitor partially attenuated the autophagy and apoptosis triggered by escin, but a ROS scavenger showed a greater inhibitory effect. Finally, the therapeutic efficacy of escin against osteosarcoma was demonstrated in an orthotopic model. Overall, escin counteracted osteosarcoma by inducing autophagy and apoptosis via the activation of the ROS/p38 MAPK signalling pathway; these findings provide evidence for escin as a novel and potent therapeutic for the treatment of osteosarcoma.

Osteosarcoma is one of the most malignant primary bone tumours, primarily affecting adolescents and young adults.^1, 2^ Whereas conventional chemotherapy is an important treatment option for osteosarcoma, it has many disadvantages.^3^ For example, chemotherapeutics tend to be highly toxic to normal tissues, leading to anaemia, neutropenia, thrombocytopenia, heart damage and a series of adverse reactions, thereby decreasing the patient survival rate.^4, 5^ Therefore, new agents with fewer side effects and better therapeutic benefits are needed.

Escin, the main active compound of horse chestnuts,^6^ has been widely used to treat swelling, exudation, and inflammation.^7^ Recently, accumulated experimental evidence has shown that escin is a potential cancer chemotherapeutic and has elucidated some of the underlying mechanisms.^8, 9, 10^ However, the principal mechanism underlying its anti-tumour effect is not fully understood. Furthermore, the effects of escin effect on human osteosarcoma and its underlying mechanisms have not been reported.

Apoptosis is the classic mechanism by which chemotherapy agents induce cell death;^11^ however, numerous studies have shown that these drugs also induce autophagy.^12^ Autophagy is usually considered to be a critical process by which cells recycle ineffectual cellular components to maintain homeostasis. This process plays a critical role in programmed cell death type II, which can be exploited to suppress tumour growth.^13^ Paradoxically, autophagy also serves as a cell survival pathway.^14, 15^ Previous studies^16, 17^ have demonstrated the dual roles of autophagy in neoplastic development; it can function as a suppressor of early-stage carcinoma and a promoter of later stage disease.^18^ Hence, the relationship between apoptosis and autophagy has not been fully elucidated, especially in the context of escin-induced osteosarcoma cell death.

Increasing evidence supports that mitogen-activated protein kinases (MAPKs) mediate apoptosis and autophagy in response to chemotherapy.^19, 20^ MAPKs are key players in the regulation of cell proliferation and cancer development. Activation of p38 MAPK could augment the processes of apoptosis and autophagy.^21^ Meanwhile, MAPK signal transduction cascades could be regulated by reactive oxygen species (ROS).^22, 23, 24^

Intracellular redox status has been reported to be associated with carcinogenesis and antineoplastic processes.^25, 26^ ROS are small and highly reactive molecules that play key roles in regulating physiological processes and maintaining redox balance.^27^ Although an appropriate amount of intracellular ROS conditions can improve cell proliferation. Recent studies^28, 29^ have reported that excessive amounts of ROS cause oxidative damage to cells, especially malignant cells, by inducing autophagy and apoptosis. Accordingly, targeting relevant signalling pathways, especially the ROS and p38 MAPK pathways, may be a good choice for osteosarcoma treatment.

In the current study, we demonstrated that escin has anti-tumour potential against osteosarcoma. We further investigated the underlying mechanisms, and the results suggest that escin may induce apoptosis and autophagy in osteosarcoma through the ROS/p38MAPK pathway.

## Results

### Escin has an anti-proliferation effect on osteosarcoma

**Figure 1:**
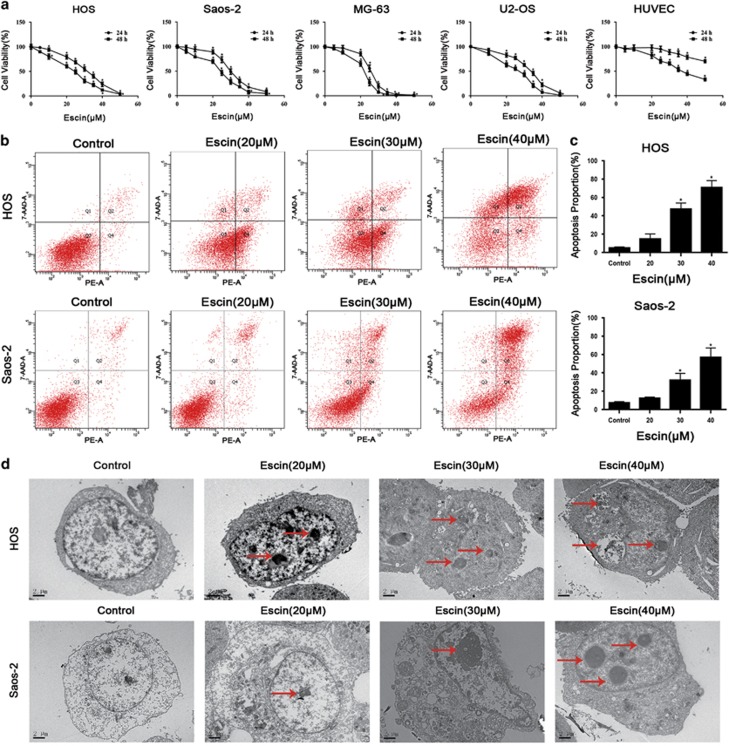
Escin inhibits human osteosarcoma cell proliferation through apoptosis. (**a**) Four osteosarcoma cell lines and HUVEC cells were treated with different concentrations of escin for 24 or 48 h. MTS assays were performed. The control group was treated with 0.1% DMSO. The data are presented as the mean of six replicates, and each experiment was performed three times. (**b**,**c**) The HOS and Saos-2 osteosarcoma cell lines were incubated with escin or control for 24 h, stained with an Annexin V-PE/7-AAD kit and analysed by flow cytometry. The proportion of apoptotic cells is presented as the mean±S.D. of three replicate experiments. (**d**) Apoptotic morphology was observed by transmission electron microscopy. The red arrows indicate apoptotic bodies and nuclear condensation. Scale bar, 2 *μ*m. *P<0.05 compared with control

To investigate the effect of escin on tumour growth, four osteosarcoma cell lines MNNG/HOS, Saos-2, MG-63 and U2-OS were exposed to different concentrations of escin for 24 or 48 h. Then, cell viability was evaluated using the MTS assays (Figure 1a). The IC50 of escin at 24 h was 30.44 *μ*M for HOS cells, 29.93 *μ*M for Saos-2 cells, 25.51 *μ*M for MG-63 cells, and 32.40 *μ*M for U2-OS cells. Moreover, in the previous studies,^30^ escin shows cytotoxic effects on control cell especially after treatment with escin at 48 h. Furthermore, our study showed that escin has less cytotoxic effects on control cell at 24 h than 48 h, so 24 h treatment is beneficial for cancer studies. The IC50 value of escin for HUVEC cells was 95.81 *μ*M for 24 h and 37.92*μ*M for 48 h. These results indicated that escin could inhibit the proliferation of osteosarcoma cells in a time- and dose-dependent manner. MNNG/HOS and Saos-2 cells harbour mutant p53 and are more chemoresistant to current chemotherapeutics;^31, 32^ these cells were used in subsequent assays. We chose concentrations of 20, 30 and 40 *μ*M escin and a timepoint of 24 h for the following experiments based on data from previous studies.^33, 34, 35^

### Escin induces apoptosis in osteosarcoma cells

**Figure 2:**
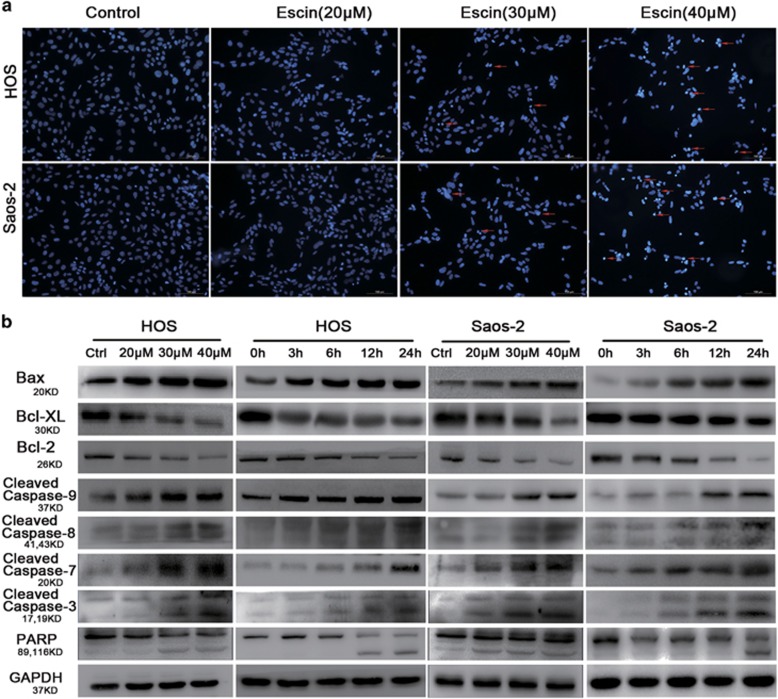
Escin induced the apoptosis of osteosarcoma cells. (**a**) Hoechst 33258 staining was used to evaluate apoptotic morphology by fluorescence microscopy. The red arrows indicate apoptotic chromatin condensation and DNA fragmentation. Scale bar, 100 *μ*m. (**b**) Osteosarcoma cells were exposed to various concentrations of escin for 24 h. The expression of the following apoptosis-related proteins was determined by western blot analysis: cleaved PARP, caspase-3, -7, -8, and -9, and Bcl-2 family members Bcl-2, Bcl-xL and Bax

To determine whether apoptosis is responsible for escin-induced cell death, we detected apoptotic cells, apoptosis-related proteins and morphological changes. Annexin-PE/PI staining results indicated that escin induced the apoptosis of osteosarcoma cell lines (Figure 1b). The apoptosis percentage of HOS cells in 40 *μ*M group is 70.8%, which is significantly higher than 5.13% in control group. The Saos-2 cells apoptosis percentage in 40 *μ*M group is 56.93%, which is 11 times greater than the apoptosis percentage in control group. Consistent with the MTS results, escin induced the apoptosis of osteosarcoma cells in a dose-dependent manner (Figure 1c). As shown in Figure 1d, nuclear condensation and apoptotic bodies were clearly observed. Figure 2a shows the appearance of apoptotic chromatin condensation and DNA fragmentation. Overall, these results demonstrate that escin induces apoptosis in osteosarcoma cells.

### Escin induces caspase-dependent apoptosis through mitochondrial-mediated pathways

To explore the molecular mechanism of escin-induced apoptosis, we performed western blots to assess apoptosis-related proteins. As shown in Figure 2b and Supplementary figure 1, the protein levels of cleaved caspase-3, -7, -8, -9 and poly(ADPribose) polymerase (PARP) was 1.85, 1.28, 1.2, 1.53 and 2.03 times higher than control group of HOS and 2.7, 1.37, 1.41, 1.66 and 3.21 times higher than control group of Saos-2 after treatment with 40 *μ*M escin, respectively. Bcl-2 family members are known to be involved in the mitochondrial-mediated apoptosis pathway, which activates the caspase cascade.^36^ Thus, we investigated Bax, Bcl-XL and Bcl-2 key members of the Bcl-2 family. Escin of 40 *μ*M group upregulated Bax protein levels (1.46 times higher than control group in HOS and 2.61 times higher than control group in Saos-2) and downregulated the expression level of Bcl-XL (decreasing 63% than control group in HOS and 51% than control group in Saos-2) and Bcl-2 (decreasing 39% than control group in HOS and 53% than control group in Saos-2) (Figure 2b and Supplementary figure1). These results indicate that escin induces caspase-dependent apoptosis through mitochondrial-mediated pathways.

### Escin induces autophagy in osteosarcoma cells

**Figure 3:**
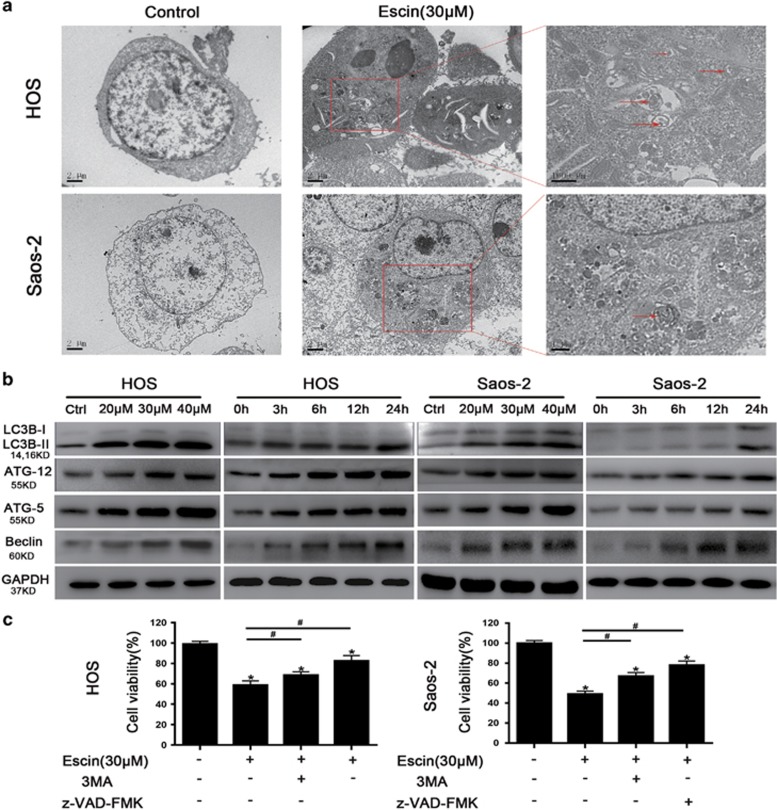
Escin induced the autophagy of osteosarcoma cells. (**a**) Morphological changes associated with autophagy were observed by transmission electron microscopy. The red arrows indicate autophagosomes. Scale bar, 2 *μ*m. (**b**) Osteosarcoma cells were treated with escin at various concentrations for different periods of time. The expression of autophagy-related protein, including LC-3B, Beclin, ATG5 and ATG12, was determined by western blot. (**c**) Cell viability was measured using an MTS assay. The histogram contains data from three separate experiments. *P<0.05 *versus* control, ^#^*P*<0.05 *versus* escin treatment

As autophagy is involved in an alternative cell death mechanism, we explored its role in escin-induced tumour inhibition. First, transmission electron microscopy (TEM) was used to directly confirm autophagosome formation (Figure 3a). LC3 is an important regulator of autophagy that promotes autophagosome formation and ATG5 and ATG12 form a complex that is essential for autophagy.^37^ The ATG5-ATG12 complex promotes the conjugation of LC3BI to phosphatidylethanolamine to form LC3BII, which is then recruited to autophagosomal membranes.^38^ Figure 3b shows that escin upregulated the levels of LC3BII, ATG-5, ATG-12 and Beclin-1 and downregulated LC3IB levels, indicating that escin regulates autophagy. Next, we investigated whether escin-induced autophagy contributes to cell death. Cell viability was assessed in the presence of 3-MA (an autophagy inhibitor) or z-VAD-fmk (an apoptosis inhibitor). 3-MA moderately reduced escin-induced cell death, while z-VAD-fmk strongly reduced cell death (Figure 3c). These data suggest that escin-induced autophagy promotes cell death and that escin causes both apoptotic and autophagic cell death.

### Escin inhibits osteosarcoma through the p38 MAPK pathway

**Figure 4:**
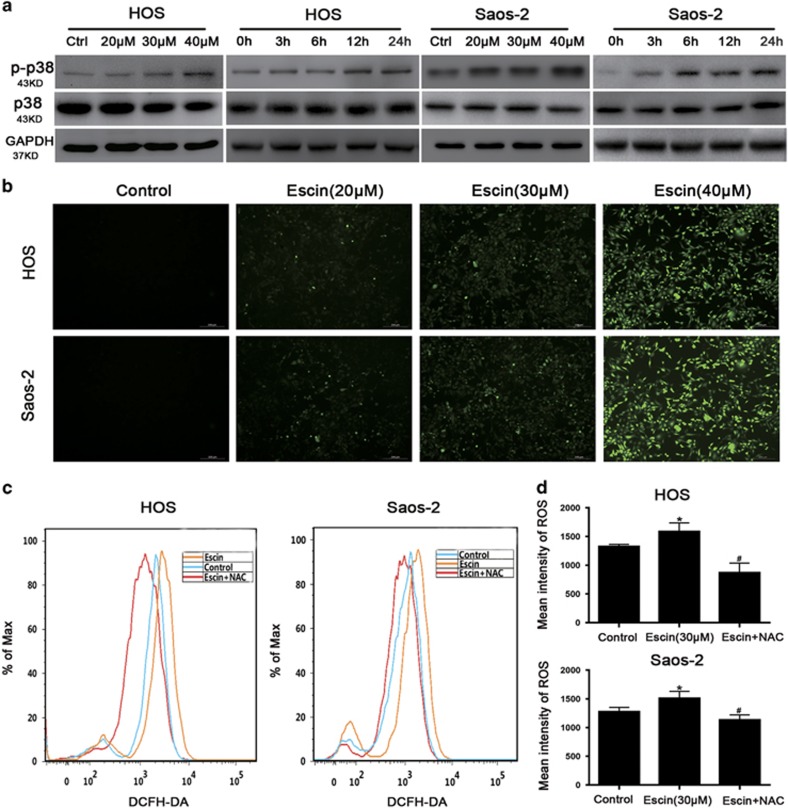
Escin triggered ROS generation and activated p38 MAPK. (**a**) Cells were exposed to escin at various concentrations for different periods of time. The levels of phospho-p38 MAPK and total p38 MAPK were determined by western blot. (**b**) Osteosarcoma cells were exposed to escin for 24 h and then stained with DCFH-DA for 30 min. ROS generation was observed by fluorescence microscopy and representative images are presented. Scale bar, 200 *μ*m. (**c**,**d**) The cells were preincubated with NAC (5 mM) for 1 h and then treated with escin for 24 h. Intracellular ROS levels were determined by flow cytometry. The histogram shows the intensity of ROS. *P<0.05 *versus* control, ^#^*P*<0.05 *versus* escin treatment

**Figure 5:**
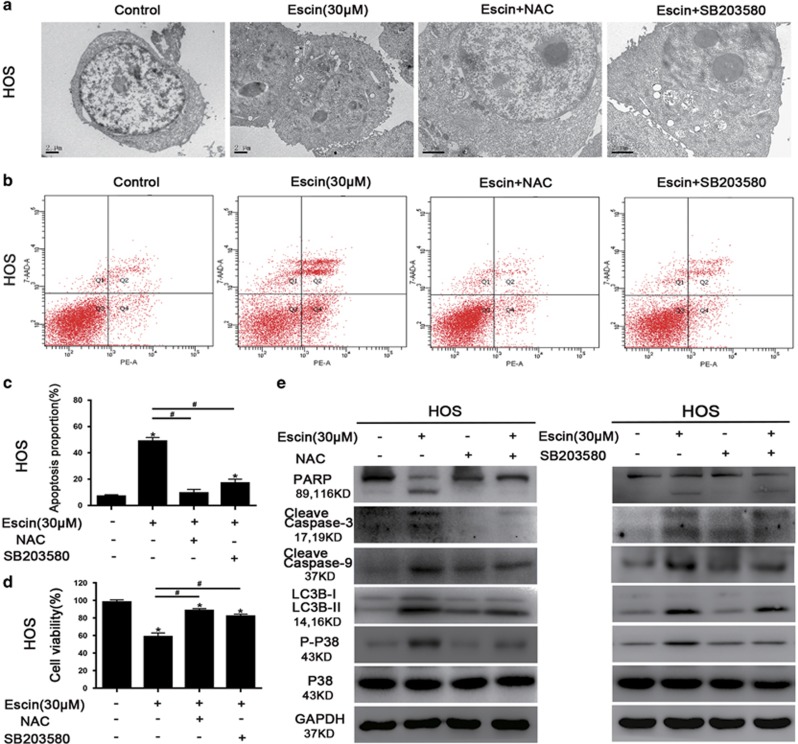
Roles of ROS and p38 MAPK in autophagy and apoptosis. Cells were precultured with the ROS inhibitor NAC (5 mM), p38 MAPK inhibitor SB203580 (10 *μ*M), autophagy inhibitor 3-MA (2.5 mM), or the apoptosis inhibitor z-VAD-fmk (20 *μ*M) for 2 h and then exposed to escin (30 *μ*M) for 24 h. (**a**) Morphological changes in cells exposed to escin and NAC or SB203580 were observed by transmission electron microscopy. Scale bar, 2 *μ*m. (**b**,**c**) The apoptotic proportion was measured by flow cytometry. The histogram includes data from three separate experiments. (**d**) Cell viability was measured using an MTS assay. The histogram presents data from three separate experiments. (**e**) The expression levels of LC3B, cleaved PARP, cleaved caspase-3, cleaved caspase-9, and p38 MAPK were analysed by western blotting. *P<0.05 *versus* control, ^#^*P*<0.05 *versus* escin treatment

The activated MAPK signalling pathway can induce cell death in cancer.^39^ Thus, we explored whether this pathway is activated by escin in osteosarcoma. The expression levels of p38, JNK and ERK-2 were determined by western blot analysis. Escin upregulated p38 expression in a dose- and time-dependent manner (Figure 4a) but had a minimal impact on JNK and ERK-2 (data not shown). Furthermore, when cells were precultured with the p38 MAPK inhibitor, the proportion of apoptotic cells and the expression of apoptosis- and autophagy-related proteins were decreased (Figures 5b–e). These results indicated that the p38 MAPK pathway is essential for escin-induced apoptosis and autophagy.

### Escin-induced ROS initiates apoptosis and autophagy in osteosarcoma cells through the ROS/p38 MAPK pathway

ROS usually plays an important role in regulating apoptosis and autophagy.^40, 41^ Thus, Escin-treated osteosarcoma cells were stained with DCFH-DA to assess ROS generation. As shown in Figures 4b and c, escin induced ROS generation in a dose-dependent manner. The ROS scavenger *N*-acetyl L-cysteine (NAC) was used to confirm the role of ROS in escin-induced cell death. NAC decreased ROS levels (Figure 4d). Less autophagic vacuoles and chromatin condensation were observed in cells pretreated with NAC than the p38/MAPK inhibitor (Figure 5a). Moreover, NAC reversed escin-induced death and apoptosis proportion to a greater extent than the p38/MAPK inhibitor (Figures 5b–d). In addition, NAC inhibited the escin-induced activation of apoptosis and autophagy-related proteins and strongly blocked p38 phosphorylation (Figure 5e). These results revealed that ROS may be the important factor upstream of p38 MAPK that initiates escin-induced apoptosis and autophagy.

### Escin inhibits osteosarcoma *in vivo*

**Figure 6:**
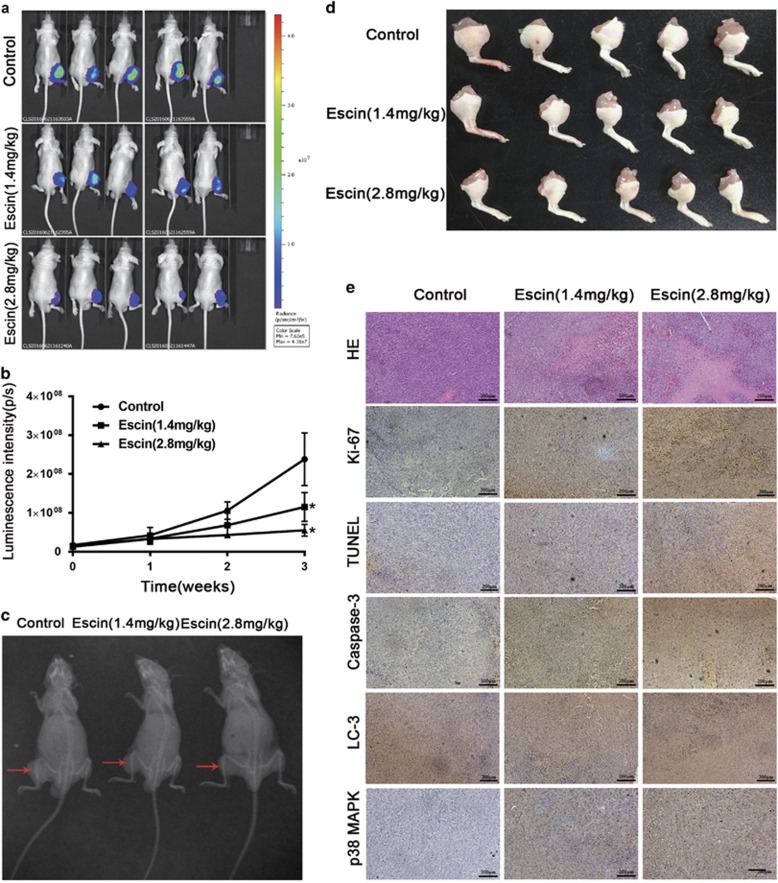
Escin inhibits the growth of human osteosarcoma xenografts *in vivo*. Saos2-luc cells were inoculated orthotopically in the right tibia of BALB/c-nu mice. After 3 days, the tumour in the tibia was assessed using an *in vivo* imaging system, and the luminescence intensity was used as an indicator of tumour size. Then, the mice were separated randomly into three groups. The next day, the mice began receiving daily intraperitoneal injections of PBS or escin (1.4 or 2.8 mg/kg). After 21 days of treatment, all mice were killed. (**a**) The tumour-located in the right leg was excised and imaged. (**b**) H&E staining was used to evaluate histology. The apoptotic status of tumour tissues was assessed by TUNEL assays and Ki-67 expression. The levels of cleaved caspase-3, LC3B and phospho-38 MAPK were further examined by immunohistochemistry. Representative images are presented. Scale bar, 200 *μ*m. (**c**,**d**) On the 21st day of dosing, the tumour in the tibia was assessed using an *in vivo* imaging system, and luciferase intensity was calculated using the *in vivo* imaging software. (**e**) The mice underwent X-ray analysis to assess osteoclasia in the tibia. The mice are shown in the following order of treatments: PBS, escin (1.4 mg/kg) and escin (2.8 mg/kg). The red arrows indicate osteoclasia. *P<0.05 compared with control

To simulate the circumstance of osteosarcoma *in vivo*, we established an *in vivo* orthotopic model of osteosarcoma by inoculating osteosarcoma cells (Saos-2 cells transferred with luciferase) into the tibia of nude mice. Tumour size was calculated based on luminescence intensity. Escin at doses of 1.4 mg/kg and 2.8 mg/kg caused a decrease in tumour luminescence intensity after 7 days of drug administration, and there was a significant difference between the two groups after 21 days (Figures 6a and b). X-ray analysis showed that escin minimized osteoclasia during osteosarcoma development (Figure 6c). The tumour-located in the right leg was excised (Figure 6d). The H&E, Ki-67 staining and terminal deoxynucleotidyl transferase-mediated (d)-UTP nick-end labelling (TUNEL) assays revealed more tumour cell death after escin treatment. The mean optical density was calculated using Image-pro software, and this immunohistochemical analysis confirmed the increased expression of LC3, caspase-3 and p38/MAPK induced by escin (Figure 6e). These results indicated that escin inhibits the growth of osteosarcoma *in vivo*.

## Discussion

Osteosarcoma is one of the most malignant neoplasms in adolescents. Current therapies incorporate surgery and combination chemotherapy, which cures nearly 70% of patients. However, the survival of metastatic osteosarcoma patients has remained virtually unchanged over recent decades, with an overall 5-year survival rate of nearly 20%.^42^ Thus, there is an urgent need to discover innovative drugs for the treatment of osteosarcoma. An increasing number of studies have shown that escin has anti-tumour potential against various types of cancer.^8, 43, 44, 45^ However, the corresponding mechanisms remain to be elucidated. In this study, we confirmed that escin inhibited osteosarcoma growth by inducing apoptosis and autophagy through the ROS/p38 MAPK pathway.

Apoptosis is the main pathway of drug-induced cell death. In this study, we found that escin activated caspase-dependent apoptosis through mitochondrial-mediated pathways, upregulated the expression of the pro-apoptotic protein Bax and downregulated the expression of the anti-apoptotic proteins Bcl-2 and Bcl-xL. Surprisingly, escin-induced cell death could not be fully reversed by the apoptosis inhibitor z-VAD-FMK, implying that apoptosis was not the only contributor.

Autophagy is involved in type II programmed cell death, particularly in apoptosis-deficient cells, and can be exploited to suppress tumour growth.^46^ In accordance with previous studies^8, 47, 48, 49^ that used TEM observation to show apoptotic and autophagic death, our study used TEM observation and western blot analysis to confirm the formation of autophagosomes and the elevated expression of autophagic markers after escin treatment, respectively. Beclin-1 is a pivotal protein in autophagy, and the upregulation of Beclin-1 can lead to autophagy.^50, 51^ We found that escin upregulated Beclin-1 expression. While many studies^52, 53^ have demonstrated that autophagy functions as a survival mechanism in cancer development, our results showed that escin-induced cell death was moderately diminished by the autophagy inhibitor 3-MA, demonstrating the contribution of autophagy to cell death instead of survival.

To further explore the upstream pathways, we tested the potential ones that might be involved in the process of apoptosis and autophagy. The p38/MAPK pathway was reported to be involved in regulating apoptosis and autophagy.^54^ Recent studies have found that higher p38/MAPK expression correlates with the increased expression of autophagic markers, such as LC3B and ATG5/ATG12, and apoptotic markers, such as caspase-3 and PARP.^21, 55, 56^ In the present study, escin induced a significant increase in p38 phosphorylation. SB203580, an inhibitor of p38/MAPK phosphorylation, was used to confirm the role of p38/MAPK in escin-induced autophagy and apoptosis. The expression levels of autophagy and apoptosis-related protein markers were decreased upon inhibition of p38/MAPK phosphorylation.

It is well documented that ROS regulate the expression of p38/MAPK.^57, 58, 59^ In the present study, increased ROS generation was observed after escin treatment. In addition, the ROS scavenger NAC strongly reversed escin-induced apoptosis and autophagy; in this context, NAC was more potent than the inhibitor of p38/MAPK phosphorylation. Taken together, we concluded that escin induced the autophagy and apoptosis of osteosarcoma cells through the ROS/p38 pathway.

It was reported previously that escin treatment resulted in significant tumour inhibition *in vivo*.^60, 61, 62^ Our *in vivo* study indicated that escin significantly inhibited osteosarcoma growth during the 3-week treatment period. Moreover, immunohistochemical analysis confirmed increased expression of LC3, caspase-3 and p38/MAPK after escin treatment. Furthermore, X-ray analysis showed that osteoclasia induced by osteosarcoma was minimized by escin.

**Figure 7:**
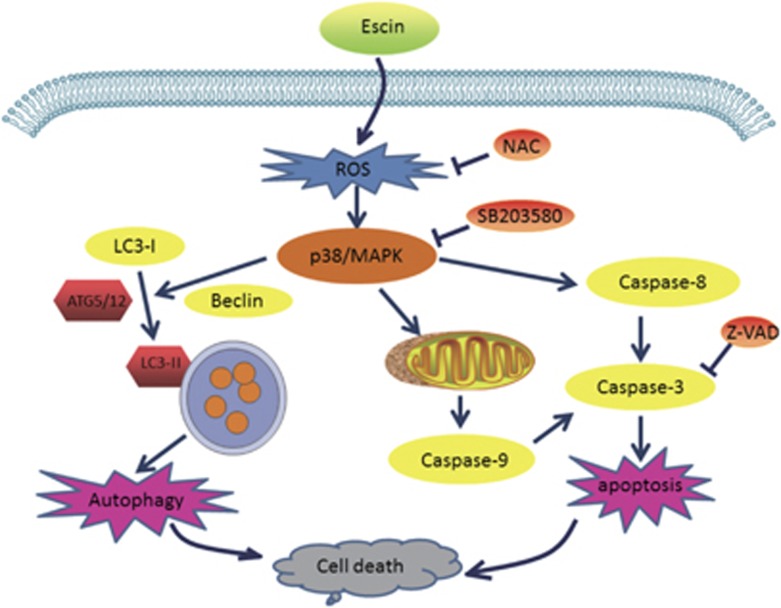
A brief diagram of the effects of escin on osteosarcoma cells

In conclusion, escin can inhibit osteosarcoma cell proliferation by inducing autophagy and apoptosis mediated by the ROS/p38 MAPK signalling pathway (Figure 7). Escin exhibited potent anti-tumour activity in the orthotopic osteosarcoma model. The results of this study provide new insights into the potential efficacy of escin in the treatment of osteosarcoma.

## Materials and methods

### Reagents and antibodies

Escin powder with purity greater than 95%, the p38 MAPK inhibitor SB203580, NAC and 3-Methyladenine (3-MA) were purchased from Sigma-Aldrich (St. Louis, MO, USA). Minimum essential medium (MEM), Eagle’s minimum essential medium (EMEM) and McCoy’s 5A Medium, RPMI 1640 Medium, fetal bovine serum (FBS), penicillin, streptomycin, PBS and 0.25% trypsin were purchased from Gibco/BRL (Gaithersburg, MD, USA). The caspase inhibitor (z-VAD-fmk) was obtained from Millipore (Billerica, MA, USA). Antibodies against PARP, caspase-3, caspase-7, caspase-8, caspase-9, Bax, Bcl-2, Bcl-XL, phospho-p38 MAPK(Thr180/Tyr182), p38, LC3B, Beclin-1, SQSTM1/p62, ATG5, ATG12 and GAPDH were obtained from Cell Signaling Technology (Beverly, MA, USA).

### Cell and cell culture

The human osteosarcoma cell lines MNNG/HOS (CRL-1547TM, ATCC), Saos-2 (HTB-85TM, ATCC), MG-63 (CRL-1427TM, ATCC), U-2OS (HTB-96TM, ATCC), HUVEC (CRL-1730TM, ATCC) were obtained from Cell Bank of Shanghai Institute of Biochemistry and Cell Biology, Chinese Academy of Sciences (Shanghai, China). According to the ATCC instructions, MNNG/HOS cells were cultured in EMEM, with MG-63 in MEM, Saos-2 in McCoy’s 5A medium, U-2OS in RPMI 1640 and HUVECs in F-12K medium. All media included 10% FBS, 100 U/ml penicillin and 100 *μ*g/ml streptomycin. The cells were incubated at 37 °C in a humidified incubator with 5% CO_2_.

### Cell viability assay

The inhibitory effect of escin on osteosarcoma cells was detected using an MTS kit (cellTiter 96 AQueous One solution Cell Proliferation Assay, Promega, Madison, WI, USA). According to the manufacturer’s instruction, cells were cultured in 96-well plates at approximately 4–5 × 10^3^ cells per well. When the cells were adherent to the bottom of the cell, the cells were treated with various concentrations of escin (0–50 *μ*M) for different periods of time (0–48 h). Then MTS was added and incubated for 2–4 h at 37 °C. The absorbance at 490 nm was measured using a MR7000 microplate reader (Dynatech, NV, USA). The data were calculated from the mean of six replicates, each experiment was conducted in triplicate.

### Morphological apoptosis

Morphological changes of apoptosis were measured by fluorescence microscopy using Hoechst 33258 staining. Briefly, cells were treated with different concentrations of escin for 24 h and then exposed to Hoechst 33258 for approximately 10 min. Then, the cells were washed with PBS twice. Fluorescence microscopy (Olympus, Tokyo, Japan) was used to visualize morphological changes, such as chromatin condensation.

### Transmission electron microscopy detection

TEM was used to observe the cell ultra-structure. Changes regarding apoptotic body and nuclear condensation indicated apoptosis, and the formation of autophagosome indicated autophagy. Cells treated with escin were first fixed with 2.5% glutaraldehyde and subsequently fixed with 1% osmium tetroxide. Cells were embedded in Epon after dehydrated with different concentrations of alcohol. Ultrathin sections (0.5 *μ*m) were generated for observation under a transmission electron microscope.

### Apoptosis measurement

Apoptosis was measured using the reagent Annexin V-PE/7-AAD kit (BD Biosciences, San Diego, CA, USA). Cells were incubated in six-well plates at approximately 2–3 × 10^5^ cells per well and treated with different concentrations of escin for 24 h. Then, the cells were incubated with Annexin V-PE/7-AAD kit for approximately 15 min at room temperature in the dark. After washing with PBS twice, the cells were resuspended in 500 *μ*l of PBS. Then, samples were evaluated using a flow cytometer (FACSCalibur, BD, San Jose, CA, USA).

### Measurement of ROS

ROS were detected by ROS assay kits (Beyotime Biotechnologies, Jiangsu, China) containing DCFH-DA. According to the manufacturer’s introduction, cells treated with escin were collected at a concentration of 1–2 × 10^6^/ml. Then, the cells were administered DCFH-DA (10 *μ*M) and incubated at 37 °C for 20 min. Every 3–5 min, the solution was mixed by inversion. Then, ROS generation was evaluated using a fluorescence microscope (Olympus) and a flow cytometer (FACSCalibur, BD).

### Western blot analysis

Cells were treated with different concentration of escin (0–40 *μ*M) for 24 h. Then, the cells were collected and lysed with RIPA lysis buffer, which included a protease inhibitor cocktail (Sigma-Aldrich). The lysing process lasted for 30 min on ice, and the solution was vortexed every 10 min. Cell lysates were centrifuged at 13 000 × *g* for 15 min at 4 °C. Then, the supernatant was collected, and the total amount of protein was quantified using a BCA protein assay kit (Pierce, Waltham, MA, USA). Equal protein was separated by 8–12% SDS-PAGE at 80 V for 1.5 h. Then, the proteins were transferred onto 2.2 *μ*m polyvinylidene fluoride membranes (Millipore) at 250 mA for 2 h in a humid atmosphere. The membranes were blocked with 5% bovine serum albumin (Sigma-Aldrich) solution for 1 h at room temperature. Then, the membranes were incubated with the primary antibody (Cell Signaling Technology) at the recommended dilution at 4 °C overnight. After being washed with TBST for 30 min, the membranes were incubated with horseradish peroxidase (HRP)-conjugated secondary antibodies (Cell signaling technology) at room temperature for 1 h. The target bands were developed by enhanced chemiluminescence kit (Millipore).

### Xenograft orthotopic model

Male BALB/c-nu mice (4 weeks old) were obtained from Shanghai Laboratory Animal Center of Chinese Academy of Sciences. The mice were maintained under specific pathogen-free conditions. Saos-2 cells were transfected with luciferase (Saos-2-luc) for *in vivo* imaging. Saos-2-luc cells (5 × 10^6^) suspended in 50 *μ*l of PBS were injected into the right tibia of each mouse. On the third day, the luminescence intensity was measured using an *in vivo* bioluminescence imaging system. When the luminescence intensity reached 1 × 10^6^ p/s, mice were randomly divided into three groups (five mice per group). Mice with a very low or high luminescence signal were killed. Then, the mice received daily intraperitoneal injections of 100 *μ*l of PBS, 1.4 mg/kg of escin or 2.8 mg/kg of escin. The luminescence intensity was measured weekly. After 21 days of dosing, all mice were killed. The tumours were excised and fixed in 10% formalin for further analysis. All treatments were approved by the Research Ethics Committee of the Second Affiliated Hospital of Zhejiang University School of Medicine, China.

### Tumour histology

The excised tumour were fixed with 4% formalin, dehydrated in a graded alcohol series and embedded in paraffin. Tumour tissue was cut into serial sections (3 *μ*m) that were stained with H&E after deparaffinization for histological analysis.

### *In situ* TUNEL staining

A TUNEL kit (Roche Diagnostics, Mannheim, Germany) was used to evaluate apoptosis. Briefly, after deparaffinization and hydration, the sections were incubated with proteinase K (20 *μ*g/ml) for 15 min at room temperature. After permeabilization, slides were incubated with the TUNEL reaction mixture. HRP-conjugated Fab fragments were used to detect labelled DNA strand breaks.

### Immunohistochemistry analysis

For immunohistochemical analysis, sections were deparaffinized in xylene and hydrated with gradient increased alcohol. To block endogenous peroxidase activity, the sections were treated with 3% H_2_O_2_ for 15 min. Next, sections were immersed in boiling sodium citrate buffer (pH 6.0) for 10 min. Then, the slides were treated with 10% goat serum for 15 min at room temperature and incubated with antibodies against cleaved caspase 3, Ki-67, LC3-B, and phospho-p38 MAPK at 4 °C overnight. After washing five times with PBS, the slides were incubated with biotin-labelled secondary antibody at 37 °C for 30 min. Immunoreactivity was detected by SABC method. A DP70 CCD camera (Olympus) couple with an AX-70 microscope (Olympus) was used to record images. Image-pro-plus (version. 6.0, Media Cybernetics) was used for digital image analysis. The measure parameters contained mean density, total area and IOD. The optical density was calibrated and the area of interest assigned value for hue, 0–30; saturation, 0–255; intensity, 0–230. Then the image was transformed into a grey-scale image, and values were measured.

### *In vivo* bioluminescence assay

For *in vivo* imaging, 200 *μ*l of luciferin (15 mg/ml) was injected intraperitoneally into the mice. After approximately 10 min, the mice were anaesthetized with isoflurane. *In vivo* imaging was performed using an IVIS 200 imaging system, and the results were analysed with Living Image Software (Version 3.0.4, Xenogen, Hopkinton, MA, USA). Total flux of the region of interest was measured in photons (p)/s for each mouse.

### X-ray

X-rays were utilized to identify the tumour- associated osteoclasia in the tibia. The mice with an orthotopic tumour underwent X-ray analysis. X-rays were taken at 40 kV and 50 mA using a high-frequency mobile C arm X-ray machine (PLX7000).

### Statistical analysis

The quantitative data were described as the mean±S.D. The data differences were calculated using a one-way ANOVA and Student’s *t*-test. All statistics were analysed with the SPSS software (21.0, SPSS, Chicago, IL, USA). Statistical significance was identified as *P*<0.05.
