# Supplementary figures and images for: Escin induces caspase-dependent apoptosis and autophagy through the ROS/p38 MAPK signalling pathway in human osteosarcoma cells in vitro and in vivo
Source: Cell Death Dis. 2017 Oct 12;8(10):e3113–. doi: 10.1038/cddis.2017.488 (PMC5682655; doi:10.1038/cddis.2017.488)

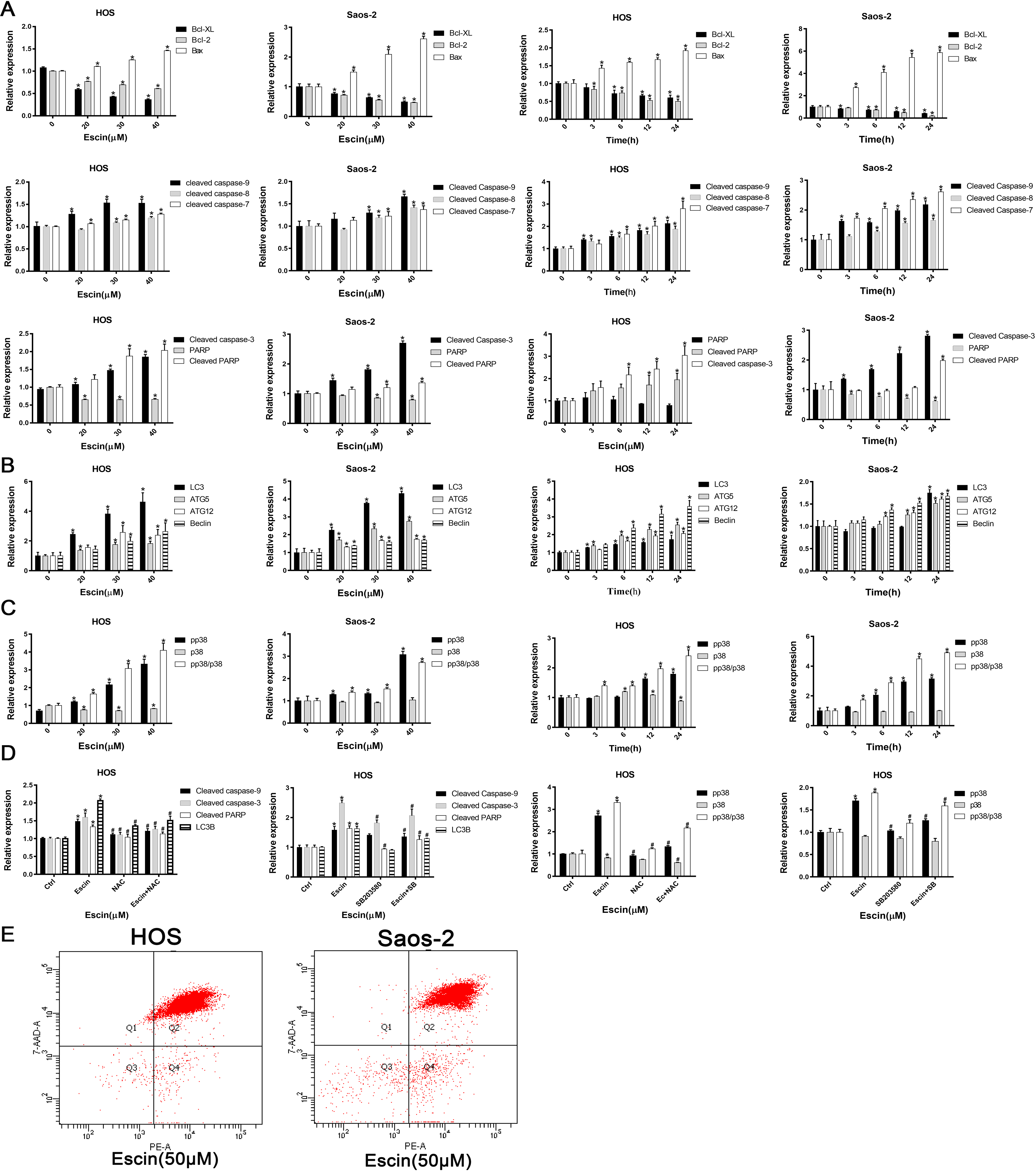

Supplement: Supplementary Figure 1 [file cddis2017488x1.tif]
