# Supplementary material for: Escin induces caspase-dependent apoptosis and autophagy through the ROS/p38 MAPK signalling pathway in human osteosarcoma cells in vitro and in vivo
Source: Cell Death Dis. 2017 Oct 12;8(10):e3113–. doi: 10.1038/cddis.2017.488 (PMC5682655; doi:10.1038/cddis.2017.488)
Supplement: Supplementary Figure Legend [file cddis2017488x2.doc]

**Supplemental Figure 1.** (A) The fold-change in protein expression levels of the apoptosis-related proteins shown in Figure2. (B) The fold-change in protein expression levels of the autophagy-related protein shown in Figure3. (C) The fold-change in protein expression levels of p38 MAPK shown in Figure4. (D) The fold-change in protein expression levels of apoptosis-related protein and autophagy-related proteins exposed to inhibitors shown in Figure5. (E) The apoptosis proportion of MNNG/HOS and Saos-2 cells exposed to 50 μM escin was measured by flow cytometry. *P<0.05 versus control, ^#^P<0.05 versus escin treatment.
